# GIS-based flood hazard mapping using relative frequency ratio method: A case study of Panjkora River Basin, eastern Hindu Kush, Pakistan

**DOI:** 10.1371/journal.pone.0229153

**Published:** 2020-03-25

**Authors:** Kashif Ullah, Jiquan Zhang

**Affiliations:** 1 Institute of Natural Disaster Research, School of Environment, Northeast Normal University, Changchun, China; 2 State Environmental Protection Key Laboratory of Wetland Ecology and Vegetation Restoration, Northeast Normal University, Changchun, China; 3 Key Laboratory for Vegetation Ecology, Ministry of Education, Changchun, China; Universiti Sains Malaysia, MALAYSIA

## Abstract

Flood is the most devastating and prevalent disaster among all-natural disasters. Every year, flood claims hundreds of human lives and causes damage to the worldwide economy and environment. Consequently, the identification of flood-vulnerable areas is important for comprehensive flood risk management. The main objective of this study is to delineate flood-prone areas in the Panjkora River Basin (PRB), eastern Hindu Kush, Pakistan. An initial extensive field survey and interpretation of Landsat-7 and Google Earth images identified 154 flood locations that were inundated in 2010 floods. Of the total, 70% of flood locations were randomly used for building a model and 30% were used for validation of the model. Eight flood parameters including slope, elevation, land use, Normalized Difference Vegetation Index (NDVI), topographic wetness index (TWI), drainage density, and rainfall were used to map the flood-prone areas in the study region. The relative frequency ratio was used to determine the correlation between each class of flood parameter and flood occurrences. All of the factors were resampled into a pixel size of 30×30 m and were reclassified through the natural break method. Finally, a final hazard map was prepared and reclassified into five classes, i.e., very low, low, moderate, high, very high susceptibility. The results of the model were found reliable with area under curve values for success and prediction rate of 82.04% and 84.74%, respectively. The findings of this study can play a key role in flood hazard management in the target region; they can be used by the local disaster management authority, researchers, planners, local government, and line agencies dealing with flood risk management.

## 1. Introduction

Flood is the most prevalent and devastating natural disaster among all natural disasters that have adverse impacts on human health, natural and artificial environments [1,2]. Flood is a major risk to human life (loss of life, injury), assets (agriculture area, yield production, homes, and buildings), communication systems (urban infrastructure, bridges, roads, and railway lines), culture heritage, and ecosystems [1–3]. Literature indicates that more than 2000 deaths occur every year due to flooding, and more than 75 million people are adversely affected in one way or another across the globe [2,3]. Many factors, including both natural and anthropogenic are responsible for catastrophic flood incidents. Flood occurs due to heavy rainfall or snow melt that overflows to adjacent areas, or flood plains, and temporarily inundates the surrounding areas [4,5]. Recent studies, indicating that climate change is a fundamental factor that induces flood in various parts of the world [6,7], Charlton et al. [8] indicate that flood disasters in a region can be considerably influenced by changes in land use patterns forming an impermeable surface, which may increase flow velocity. Aside from these, many other factors that trigger flood occurrence are: slope, elevation, land use, curvature, Normalized Difference Vegetation Index (NDVI), proximity to rivers, etc., [9,10]. Due to the complex nature of floods, their frequent occurrence and extensive destruction across the globe, a large number of scientists have devoted significant effort to investigate and understand flood hazard for better mitigation and management [4,11–14].

Flood is a natural phenomenon and its complete prevention is not possible; however, the risk of the flood can be minimized by appropriate planning and mitigation measures. Flood management is one of the key steps in mitigation and risk reduction. Various studies have indicated that identification of flood risk zones and application of essential risk reduction measures (structural and non-structural) can effectively reduce flood losses to an acceptable level [14,15]. Moreover, flood hazard mapping plays a significant role in flood planning, early warning systems, emergency response services, and design of flood risk reduction measures [14,16]. So far, various studies have been conducted to assess and map flood-prone areas in different regions of the world [9,17,18]. The study of Guo et al.[14] stated that the scope of conventional approaches for flood hazard mapping is usually narrow, due to a lack of sufficient data. For example, rainfall-runoff modeling methods, watermarks on buildings, models involving numerical simulations, etc., are not appropriate for comprehensive river and flood analysis [2,10]. The acquisition of adequate data for flood mapping using these methods and similar conventional techniques is expensive, time-consuming, and often not available at the watershed or regional level, especially in developing countries. Today, remote sensing and GIS are powerful tools and provide different data sources for hazard management, flood susceptibility, and its forecast [7,11,19].

Over the past few decades, numerous methods have been developed and used to investigate flood hazard and risk assessment. These methods include the analytical hierarchy process (AHP) [13,19], fuzzy logic and genetic algorithms [17], variable fuzzy theory [14], hydrological forecasting systems [20,21], random forest [22], artificial neural networks (ANNs) [18,22], adaptive neuro-fuzzy interface systems [23], logistic regression [24], weight of evidence [25,26], analytic network process (ANP) [27], statistical index [28], Shannon’s entropy [29], Copula-Based Bayesian Network [30], and frequency ratio models [1,25,31]. The ANN approach, which has been used for flood susceptibility mapping [18,32], tries to make an association between some input factors and an outcome. However, Tiwari and Chaterjee [33] reported that the length of the dataset can cause errors in the process of ANN modelling and also poor prediction. Das [12] applied AHP to map flood hazard zonation in the Vaitarna basin, Maharashtra, India. However the drawback of AHP lies in its dependence on expert opinion [34]. The most common statistical methods of logistic regression and frequency ratio (FR) can be considered as significant methods that use a simple and understandable perception [1,25,26,35]. Tehrany et al. [9] reported that logistic regression and FR models can generate acceptable flood risk maps, and the process of analysis is easily understandable. Among bivariate statistical models, the FR model is considered one of the most important method that is easy to apply and can produce acceptable risk analysis and mapping [9,26,35,36]. Accordingly, FR was selected from the set of bivariate statistical methods for this study. The results obtained from this model are easy to interpret. Although this model is infrequently used in flood hazard mapping, its superior performance has been proven in other fields of natural hazard such as landslides [34,37–40]. Furthermore, some studies show that bivariate statistical models sometimes have a higher accuracy than machine learning models, which require huge amounts of data as training for better accuracy [40–42]. FR is the bivariate statistical method that can consider the correlation between dependent factors (historical flood points) and independent factors (flood-causative factors) [1,25,43]. FR models have been successfully applied to flood susceptibility and vulnerability assessments in different flood prone regions of the world [1,25,26].

The Panjkora River Basin (PRB) is located in the eastern Hindu Kush region, Khyber Pakhtunkhwa province, Pakistan, which experiences flood events almost every year, generally during the monsoon seasons (June–September) [44]. Over the last decade, many disastrous floods have occurred in the region, which negatively affected human lives, property, agriculture, and other infrastructure [45–47]. The most devastating flood events have been recorded in the years 2005, 2010, 2014 and 2016. It has been reported by Rahman and Dawood [48] that climate change has intensified the spatiotemporal variability of rainfall, which poses serious threats to the local communities in the form of floods. In addition, the complex topography of the region coupled with the fragile socioeconomic condition of the local people triggers flood risk in the region [46]. So far, few studies have been conducted to assess flood hazards and map the flood-prone zones, especially in the middle and lower catchment of the PRB [46,47]. Therefore, the present study was designed to map the flood-prone areas in PRB and propose effective measures for flood risk reduction in the study region. The study is based on an integrated approach using ground-based observation, remote sensing, and relative frequency ratio (RFR) techniques. The current study is the first of its kind to map the flood-prone areas in the PRB using the RFR model.

## 2. Materials and methods

### 2.1 Description of the study area

**Fig 1:**
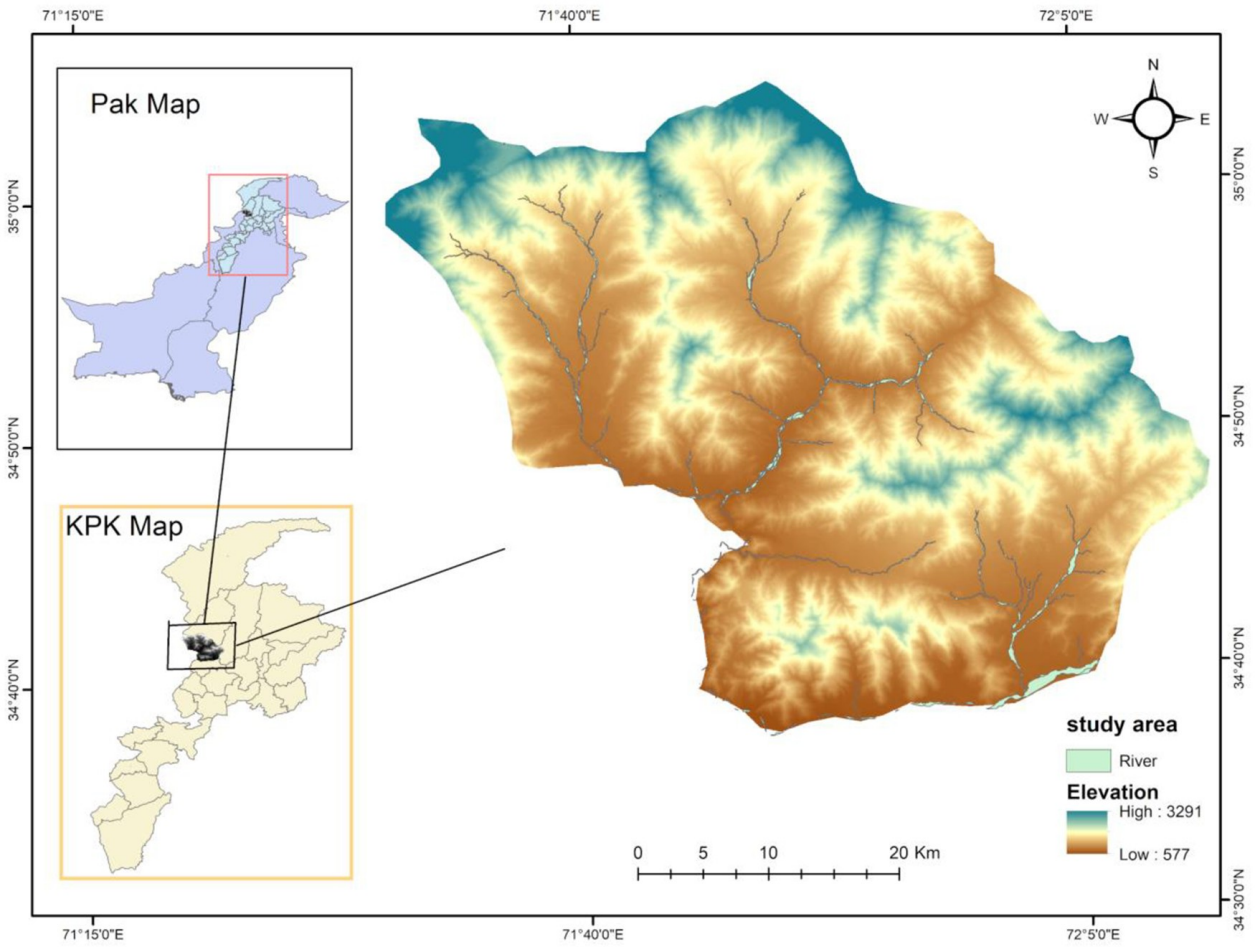
Study area.

The study area is located in the eastern Hindu Kush Khyber, Pakhtunkhwa province, Pakistan with the geographical extent of “34.33°–35.0° N latitudes and 71.0°–72.0° E longitudes” (Fig 1). It covers the lower and middle catchments of the PRB, and comprises an area of 1,741 km^2^. A river runs through it northeast to southwest, joining up with tributaries and finally draining into the river Swat at Qalangi village [46]. Climatically, in winter, the temperature drops to -12 °C while in summer, the temperature rises to 35 °C. In monsoon seasons (June–September), the PRB receives more than 800 mm of rainfall [47]. In the study area, the soil structure varies from a clayey nature to loam and sandy loam. In most places, due to steep and delicate slopes, the ground is exposed and vulnerable to erosion. The fertile soils exist mostly on moderate slopes. Such areas are commonly used for agriculture.

In recent years, the study area experienced disastrous floods in 2005, 2010, 2014, and 2016 with adverse impacts on people lives, property, agriculture, and infrastructure [46,47]. During the summer season, heavy rainfall causes floods in the region, and sometimes the extraordinary activity of the monsoon causes high surface run-off and peak discharge.

### 2.2 Flood inventory mapping

**Fig 2:**
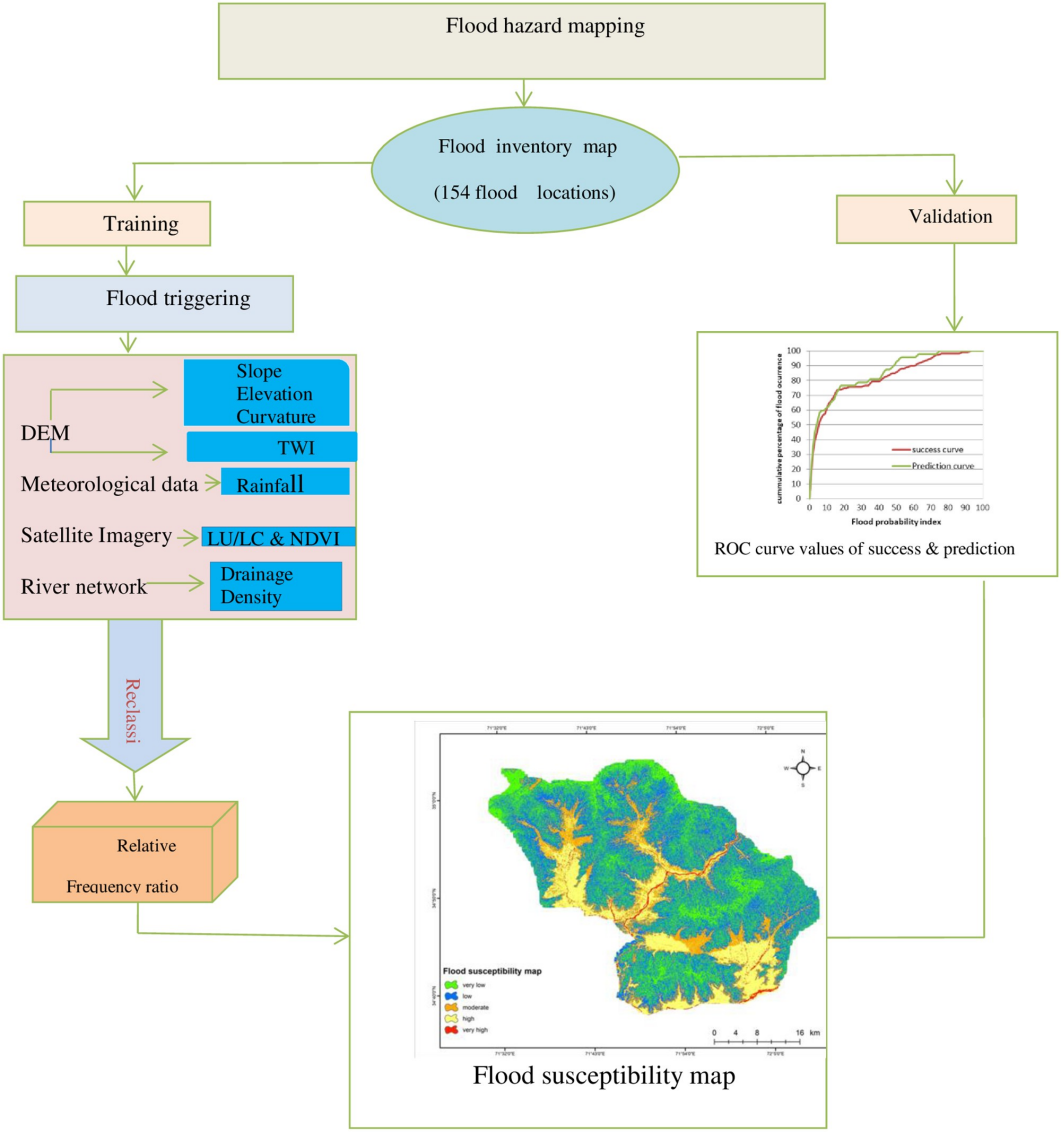
Historical floods inventory map.

The database of past floods is important to the study of the relationship between different flood triggering factors and flood occurrence [18,49]. Moreover, the accuracy of the flood susceptibility mapping greatly relies on the accuracy of previous floods events [7,25,49]. In the present study, the flood inventory database was created after identifying 154 flood points using existing flood reports of the National Disaster Management Authority, Pakistan, Provincial Disaster Management Authority, Khyber Pakhtunkhwa, field surveys, and interpretation of satellite and Goggle earth images before and after the 2010 devastating flood in the target area. Based on the literature reviews, 70% of flooded locations (107 locations) were selected randomly as a training dataset to prepare the flood hazard map and 30% of the locations (47 locations) were used for validation of the results (Fig 2) [7,26,50].

### 2.3 Identification of flood triggering and causal factors

**Fig 3:**
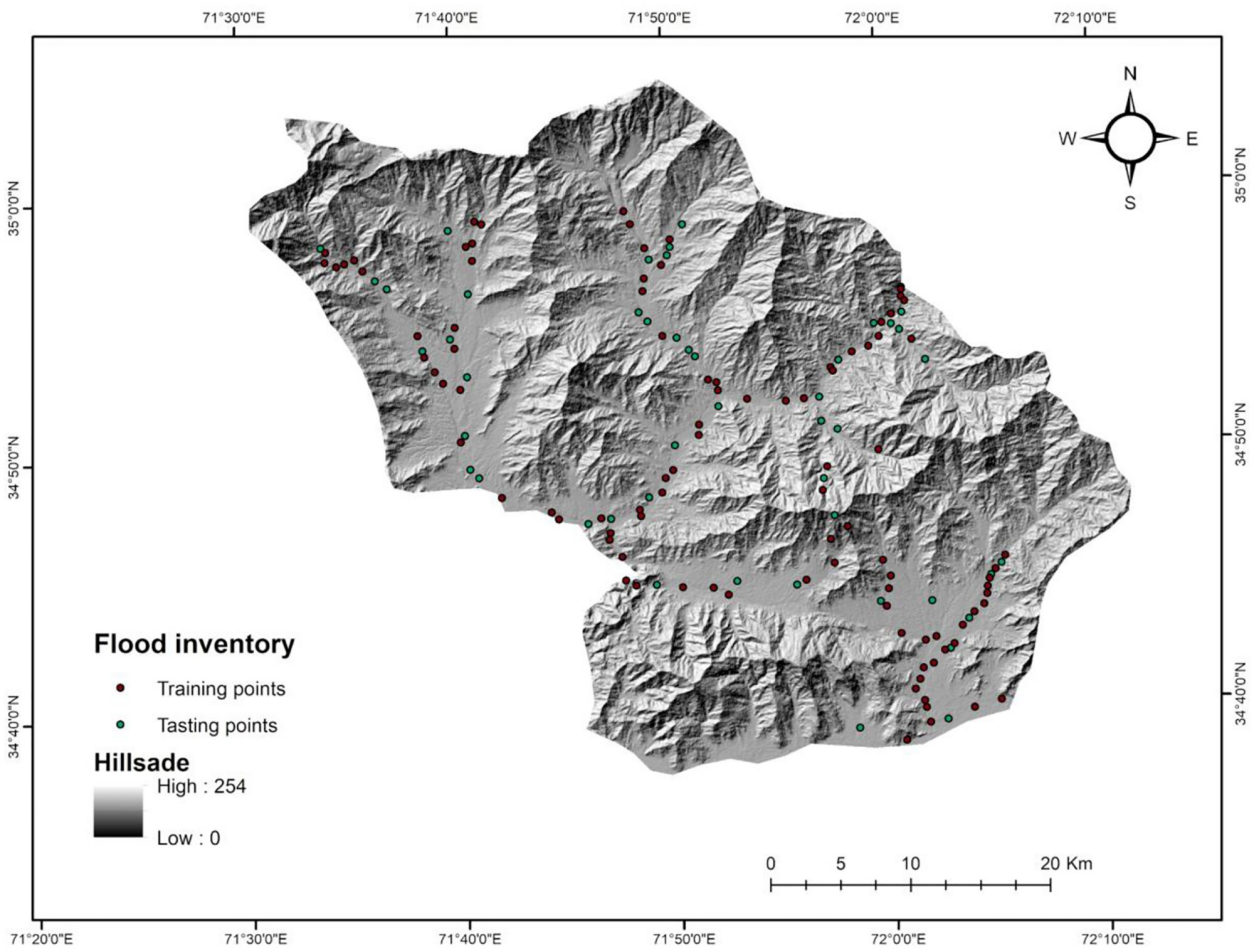
Flow chart of the methodology adopted for flood hazard mapping in PRB.

**Fig 4:**
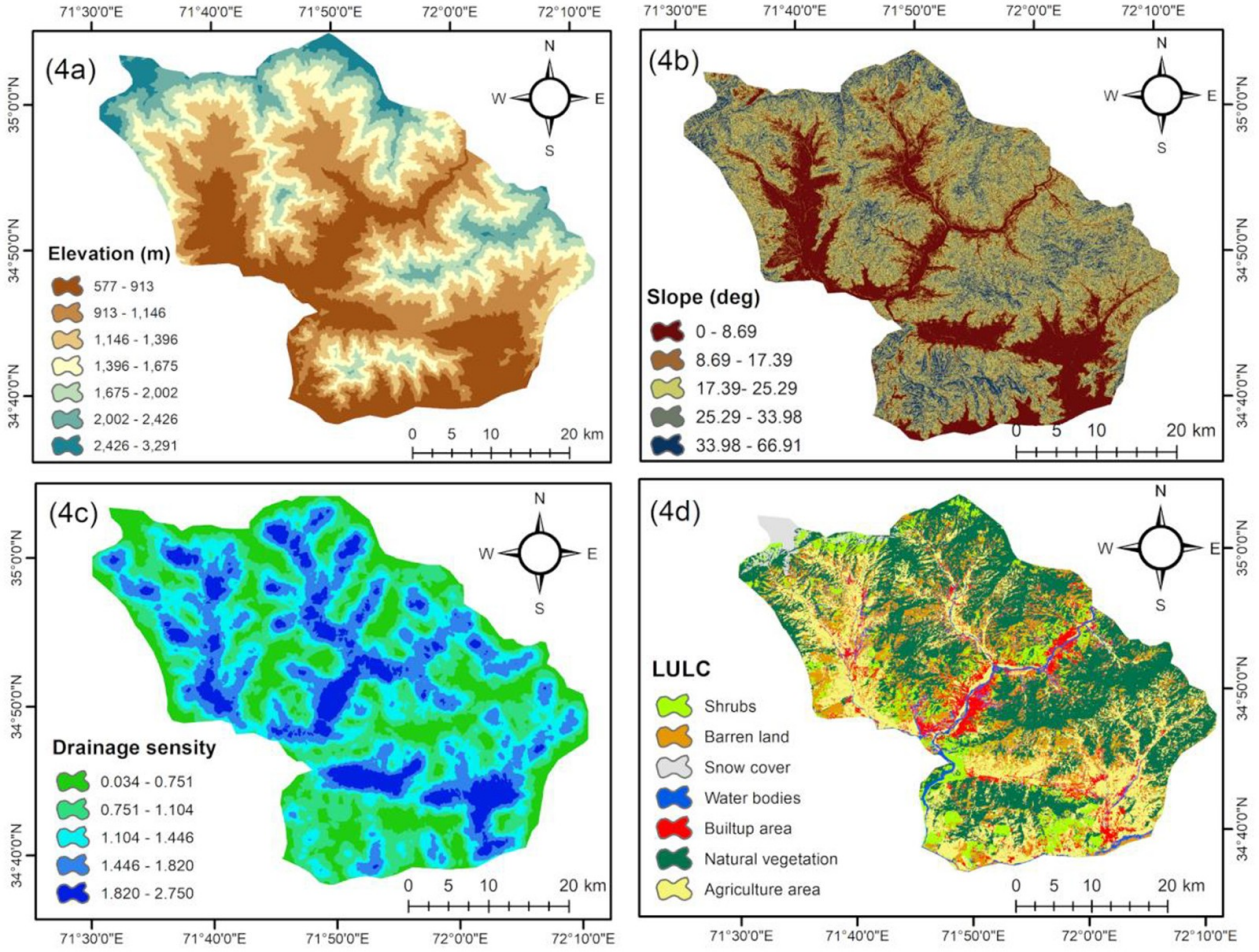
Flood conditioning factors: (a) elevation, (b) slope, (c) drainage density, (d) LULC.

**Fig 5:**
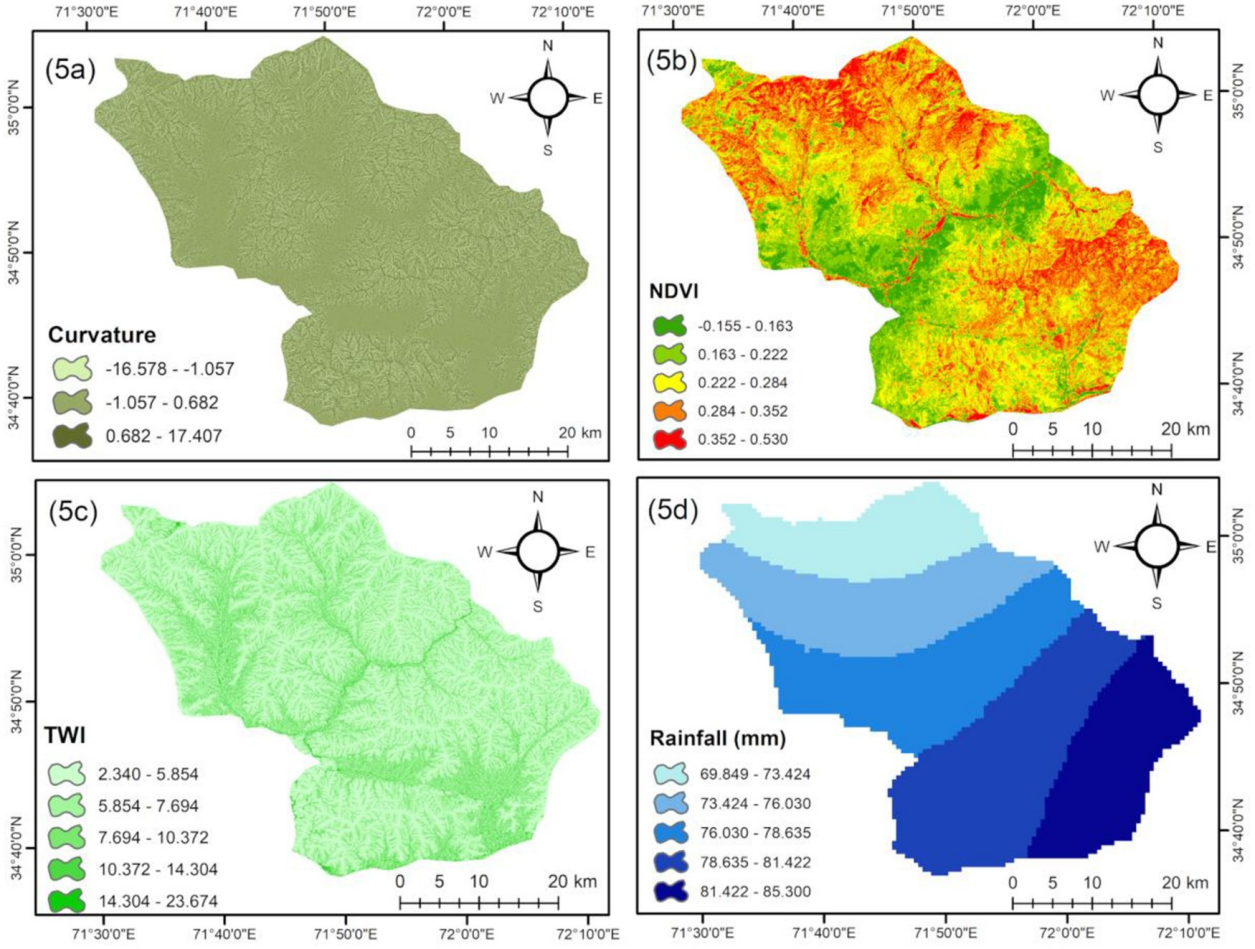
Flood conditioning factors: (a) curvature, (b) NDVI, (c) TWI, (d) rainfall.

To evaluate the flood vulnerability, it was necessary to investigate a series of flood triggering and causal factors and their relationship with flooding [51,52]. In past studies, different flood-controlling factors have been used [1,12,13]. There is no specific guideline for selecting flood-controlling factors that affect flood occurrence. The selection of flood-controlling factors is an important step for flood hazard mapping and depends on physical and natural characteristics of the study area and data availability [18,53]. The methodology adapted for this study is shown in Fig 3. To prepare the flood susceptibility map for the PRB, various satellite images and ancillary datasets were acquired from government organizations and web sources: (i) Advanced Spaceborne Thermal Emission and Reflection Radiometer Digital Elevation Model (ASTER DEM) of 30 m spatial resolution; (ii) Landsat 8 (OLI) imagery (Date: 19-September-2018) are downloaded from USGS official website (https://earthexplorer.usgs.gov); and (iii) monthly rainfall data from 1980 to 2016 collected from the Regional Meteorological Center, Peshawar. In this study, we have identified and selected eight flood causative factors, namely, slope, elevation, curvature, TWI, land use and land cover (LULC), rainfall, NDVI, and drainage density to generate thematic layers for flood hazard mapping based on a literature review and local conditions [10,13,20]. Moreover, ArcGIS (10.2), SAGA GIS, and Erdas were used to generate the required thematic layers. The relationship of each factor with flooding is discussed below in Table 1 and illustrated in Figs 4 and 5.

**Table 1:** Identification of flood triggering and causal factors.

Flood triggering and causal factors	Procedure of preparation of each factor and its relationship with flood susceptibility
Elevation	Elevation is one of the prime factors controlling floods in a region [54]. Lower and lowland areas may get flooded faster as water flows from high altitude to low regions. Areas located at a higher elevation usually have a lower probability of flooding compared to lowland areas [12,53]. In this study, the elevation map was prepared from ASTER DEM 30 m resolution and classified into seven classes using the natural break method in ArcGIS 10.2 (Fig 4a).
Slope angle	In hydrological studies, slope plays an important role; it regulates surface water flow [7,12]. Slope controls the surface runoff and the intensity of water flow that provokes erosion of soil and vertical percolation [32]. The area having a lower slope is more exposed to flooding [53]. In the PRB, the angle variation in slope ranges from 0°–68°. The slope map was directly created from ASTER DEM using the surface tool in ArcGIS 10.2 (Fig 4b).
Drainage density	Drainage density is defined as the ratio of the total length of the watershed channels to the total area of the basin [26]. Drainage density has a direct relationship with flooding. A higher likelihood of flooding is directly linked to higher drainage density as it indicates a high surface runoff [43]. The stream network was extracted from ASTER DEM and a drainage density map was developed by applying line density in spatial analyst ArcGIS (10.2). The drainage density map was classified into five classes using a natural break (Fig 4c).
Land use/land cover	Land use and land cover (LULC) are important factors in generating surface runoff and potential flooding in a watershed [55,56]. LULC directly or indirectly affects penetration, evapotranspiration, and surface runoff generation [1,54]. The LULC map was prepared from the Landsat-8 (OLI) satellite imagery (Fig 4d) through supervised classification techniques using a maximum likelihood algorithm in Erdas 2015. The LULC map was classified into seven classes: shrubs, agriculture, natural vegetation, water bodies, built area, barren land, and snow cover.
Curvature	Curvature is regarded as one of the flood-conditioning factors in most literature [9,12]. Curvature is the rate of change in slope gradient in a specific direction: the values represent the morphology of the topography [22,25]. A positive curvature means that the slope gradient is convex in the upward direction, a zero value represents no curvature, and a negative value indicates the slope is concave upward [8]. The curvature map was prepared from ASTER DEM in ArcGIS 10.12 (Fig 5a).
Normalized Difference Vegetation Index	The NDVI is another factor that is a valuable index in assessing vegetation coverage and its outcome on flooding in a basin [25]. The NDVI normally ranges from -1 to +1[7]. The NDVI values ranged from -0.15 to 0.53 in the study region. The NDVI map was prepared from a satellite image of Landsat 8 (OLI). The NDVI values were calculated using equation Eq 1 [7]. NDVI=(NIR−VIS)/(NIR+VIS)(1) where *VIS* and *NIR* are the spectral reflectance measurement acquired in the visible (red) and near-infrared region respectively (Fig 5b).
Topographic wetness index	TWI is generally used to measure the effect of topography on runoff generation and the amount of flow accumulation at any position in a river catchment [12,25]. TWI was calculated from the flowing formula; $TWI=\text{ln}\left[\frac{As}{\text{tan}\left(\beta \right)}\right]\;(2)$ where *As* is the upstream contributing area and *β* is the slope gradient. High TWI regions have a high vulnerability to flooding and lower TWI regions have lower flood vulnerability [57]. TWI has been calculated directly by processing ASTER DEM in SAGA GIS (Fig 5c).
Rainfall	In Pakistan, flooding usually occurs after heavy rainfall. Literature indicates that rainfall has a direct relationship with river discharge and a large amount of rainfall in a short time can generate flash floods in semi-arid regions [12,43,53,54]. The monthly rainfall data from 1980 to 2016 were collected from the Regional Meteorological Center (RMC) Peshawar. The rainfall distribution map has been prepared from average rainfall through Inverse Distance Weighting (IDW) Interpolation in ArcGIS 10.2 (Fig 5d).

### 2.4 Relative frequency ratio model

Flood hazard assessment is an important technique in hydrological studies. In this study, an RFR model is used to map flood prone zones in the PRB. FR is a bivariate statistical analysis method, based on the spatial distribution (probability) dependent factor (flood location) and flood triggering and causal factors (i.e., slope, elevation, etc.) [25,42].

The bivariate probability of each independent flood triggering factor was determined by its relationship with flood occurrence [1,25]. The higher the bivariate probability (greater than 1) the stronger is the correlation between flood incidence and flood triggering factors, and the lower the probability (less than 1), the weaker the correlation [1,25,50].

The FR values were calculated using (Eq 3) for all sub-classes of flood triggering factors based on their relationships with flood inventory, as shown in Table 2.

$$FR=\frac{Flood\;points\;in\;factor\;class/Total\;flood\;points}{Factor\;class\;area/Total\;area}$$ (3)

**Table 2:** Calculation results of FR and RF for all classes of factors.

Factors	Factor classes	No of points	% of points	class area	% of class area	FR	RF
Elevation	1	59400	61.11	425764	21.75	2.81	0.56
	2	16200	16.67	444873	22.72	0.73	0.15
	3	10800	11.11	377469	19.28	0.58	0.12
	4	7200	7.41	299902	15.32	0.48	0.10
	5	2700	2.78	216801	11.07	0.25	0.05
	6	900	0.93	139457	7.12	0.13	0.03
	7	0.00	0.00	53488	2.73	0.00	0.00
Slope	1	68400	70.37	466272	23.82	2.95	0.68
	2	12600	12.96	400501	20.46	0.63	0.15
	3	10800	11.11	477898	24.41	0.46	0.10
	4	4500	4.63	416184	21.26	0.22	0.05
	5	900	0.93	196899	10.06	0.09	0.02
Drainage density	1	2700	2.78	389221	19.96	0.14	0.02
	2	7200	7.41	511734	26.24	0.28	0.04
	3	18000	18.52	444233	22.78	0.81	0.12
	4	33300	34.26	417495	21.41	1.6	0.24
	5	36000	37.04	187554	9.62	3.85	0.58
LULC	1	900	0.01	184627	0.09	0.10	0.01
	2	7200	0.07	232148	0.12	0.63	0.05
	3	0.00	0.00	28053	0.01	0.00	0.00
	4	23400	0.24	55824	0.03	8.45	0.64
	5	17100	0.18	172316	0.09	2.00	0.15
	6	14400	0.15	872325	0.44	0.33	0.03
	7	34200	0.35	415251	0.21	1.66	0.13
Curvature	1	81000	8.330	329225	16.82	0.50	0.23
	2	83700	86.11	1293895	66.09	1.30	0.61
	3	54000	5.560	334632	17.09	0.33	0.15
NDVI	1	27000	27.78	210062	10.73	2.59	0.43
	2	17100	17.59	450336	23.01	0.76	0.13
	3	18000	18.52	536189	27.39	0.68	0.11
	4	17100	17.59	476438	24.34	0.72	0.12
	5	18000	18.52	284424	14.53	1.27	0.21
TWI	1	19800	20.37	817198	41.74	0.49	0.04
	2	32400	33.33	725250	37.05	0.90	0.08
	3	20700	21.30	299045	15.27	1.39	0.12
	4	18900	19.44	90685	4.63	4.20	0.37
	5	54000	5.560	25574	1.31	4.25	0.38
Rainfall	1	9000	0.10	268834	13.78	0.76	0.14
	2	19800	0.21	387366	19.86	1.17	0.21
	3	28800	0.30	450908	23.12	1.00	0.26
	4	14400	0.15	514166	26.37	0.64	0.11
	5	22500	0.24	328400	16.84	1.56	0.28

In the next step, the FR was normalized in a range of probability values [0, 1] as relative frequency (RF) using Eq 4.

$$LRF=\frac{Factor\;class\;FR}{\sum Factor\;classes\;FR}$$ (4)

After the normalization, the RF still has the drawback of considering all causative factors as having equal weight. To overcome this problem and to find the mutual interrelationship among flood causative factors, a predictor rate (PR), or weight, was calculated by rating each flood causative factor with the training data set (Eq 5) [58–60].

$$PR=\left({RF}_{max}-{RF}_{min}\right)/\left({RF}_{max}-{RF}_{min}\right)Min$$ (5)

Finally, the flood susceptibility index was obtained by the summation of the PR of each factor and the RF of each class using Eq 6.

$$FSI=\sum_{i=1}^{n}{PR}_{i}\times {RF}_{i}$$ (6)

where *PR*_*i*_ is the weight of each triggering factor, *RF* is the class weight of each subclass of flood triggering factor, and *n* is the number of factors. In this study, *n* = 8.

## 3. Results and discussion

In this study, the flood susceptibility of the PRB has been assessed by using an integrated approach of the bivariate statistical method (FR) with geospatial techniques. FR was used to calculate the correlation between flood occurrence and flood triggering factors. Table 2 shows the relationship between different flood causative factors, sub-classes, and flood occurrence in the PRB. Eight flood-triggering factors, namely, elevation, slope, drainage density, LULC, curvature, NDVI, TWI, and rainfall were used in the study. There is a direct positive relationship between FR and flood probability.

Elevation is an important factor of flood occurrence, as water always flows from higher locations to low land areas [52]. The elevation class 577–913 m has the maximum RF value of 0.56, followed by 913–1146 m and 1146–1675 m with RF values of 0.15 and 0.12, respectively. The analysis reveals that almost 65% of past floods occurred in the first three classes of elevation. Elevations higher than 2436 m have the lowest RF value (0.00, see Table 2). These results are in agreement with previous studies, which found a low probability of flood occurrence at higher elevated regions and a high probability of flooding in lowland areas [54,57].

Slope regulates the incidence of flooding, as lowland areas in the rainy season have a strong connection with the flood state. It has been reported that a lower slope gradient has more chances of flooding and flood events [51,56]. The infiltration process is also partly controlled by the slope gradient. An increasing gradient decreases the process of infiltration but increases the surface runoff; as a result, in regions having a sudden descent gradient, an enormous extent of water becomes stagnant and causes flood conditions [61]. The results show that the two lower slope gradient classes, i.e., <6.8° and 6.8°–15.4° have the highest RF value of 0.68 and 0.15, respectively. In contrast, the slope gradient above 29.4° shows the lowest RF value of 0.02 (Table 2). Approximately 68% of fast floods occurred in PRB areas having slope lower than 25°. Fig 4b indicated that the lower slope gradients are pointed on both sides of the river.

Drainage density is considered an essential element of flooding. The higher likelihood of flooding is strongly linked to higher drainage density as it points toward a greater surface runoff [54]. In this study, the drainage density has a direct relationship with flooding. The probability of flooding increases with an increase in drainage density and decreases with a decrease in drainage density. Drainage density was divided into five classes using the natural break method (Fig 4c). The class 1.82–2.75 km/km^2^ and 0.034–0.75 km/km^2^ have the highest and lowest probability of flooding with RF values of 0.58 and 0.2, respectively (Table 2). High drainage density refers to high surface runoff, therefore, high flood probability exists in areas having high drainage density [43,54].

Land use patterns reveal the type of utilization of land by people and natural processes [7,12]. Urban areas increase runoff due extensive impervious soil and fallow farmland increases runoff where there is no vegetation cover to control and prevent the rapid flow of water to the soil surface. There is risk of flooding and soil erosion in those areas; therefore, they are the most vulnerable areas to flooding. For LULC, the maximum weights were allocated to water bodies (RF = 0.61), followed by built-up areas (0.15) and agriculture areas (0.13), while forest and snow cover are least vulnerable areas in the region with RF values of 0.00 and 0.3, respectively (Table 2). Built-up areas located in proximity to rivers are most vulnerable to flooding due to their economic resources, infrastructure, and large population [7,12,25].

Similarly, curvature is also an important factor and represents the morphology of the topography [12,25,62]. The curvature map is classified into three classes. A positive value of curvature represents a convex surface, zero a flat surface, and a negative value a concave surface [7,54]. The results show that the highest RF was obtained for the flat surface at the rate of 0.61, while the lowest RF was obtained for the concave surface at 0.15 (Table 2). It was observed that approximately 83% past flood had occurred in flat and convex shape slopes.

The NDVI is another important conditioning factor of flooding. The index values range from -1 to +1[7]. Khosravi et al. [25] stated that the negative values show water and the positive values show vegetation so, NDVI has negative relationship with flooding: higher NDVI values indicate lower probability of flood and lower NDVI values indicate higher flood probability. In this study, the NDVI values range from -0.15 to 0.53 and were classified into five classes using a natural break method (Fig 5b). For the class -0.15 to 0.16, the RF was highest 0.43 (Table 2), which means that there is a high probability of flooding in the study region [43].

The TWI was classified into five classes: <5.85, 5.85–7.69, 7.69–10.37, 10.37–14.30, and 14.30–23.67 (Fig 5c). The RF values for the TWI classes of 14.30–23.67 and 10.37–14.30 were calculated as the highest at 0.38 and 0.37, respectively. Similarly, the RF value for the TWI class of <5.85 was lowest at 0.04 (Table 2). TWI has a direct positive relationship with flooding [12,25]. The higher TWI class refers to higher chances of flooding in the watershed [10]. The results indicate that the higher TWI was found in the south, northeast, and middle of the study area (represented with a blue color in Fig 5c), and a low TWI was mostly present in the north and in steep slopes.

Except for glaciers, rainfall is the only source of water in the study region. A sudden rainfall in an area can cause flash flood conditions in semi-arid regions [12]. A large number of previous studies have established a relationship between rainfall and flooding [17,52,54]. The PRB is characterized by semi-arid climatic conditions, where an enormous amount of rainfall occurs summer season due Asian monsoon system which causes flash flood [63]. The rainfall map was reclassified into five classes with natural breaks. The highest RF value (0.29) was observed for class >81.43 mm followed by class 76.03–78.63, 73.42–76.03, and 69.84–73.42 with RF values of 0.26, 0.21, and 0.14, respectively (Table 2). The lowest RF value of 0.11 was observed for class 78.63–81.42 mm. It is interesting to note that the class 78.63–81.42 mm is the second highest rainfall region but the least vulnerable, because this region is characterized by high elevation, high slope gradient, and dense forest and floods occur in lowland area. Therefore an increase in rainfall has no impact on flooding [25].

After the preparation of all eight layers of flood triggering and causal factors and giving weights to each parameter using FR and RF, a final hazard map was obtained by summation of each factor PR (weight) and each class RF in a raster calculator ArcGIS 10.2 environment using Eq 6. The flood hazard index (FHI) values of the study area are found to lie in the range from 8302 to 100311. The FSI values of the total area were divided in five subclasses using a natural break method: very low, low, medium, high, and very high and indicated in Fig 5. The analysis illustrates that approximately 15% of the total area is in a very high and high flood hazard zone, 14% is in medium, 42% is in low, and 29% is in safe areas (Table 3).

**Table 3:** Classification of different hazard classes.

Hazard class	Class area (sq.km)	% of Area
Very low	509	29
Low	723	42
Medium	248	14
High	240	14
Very high	21	1
Total	1741	100

**Fig 6:**
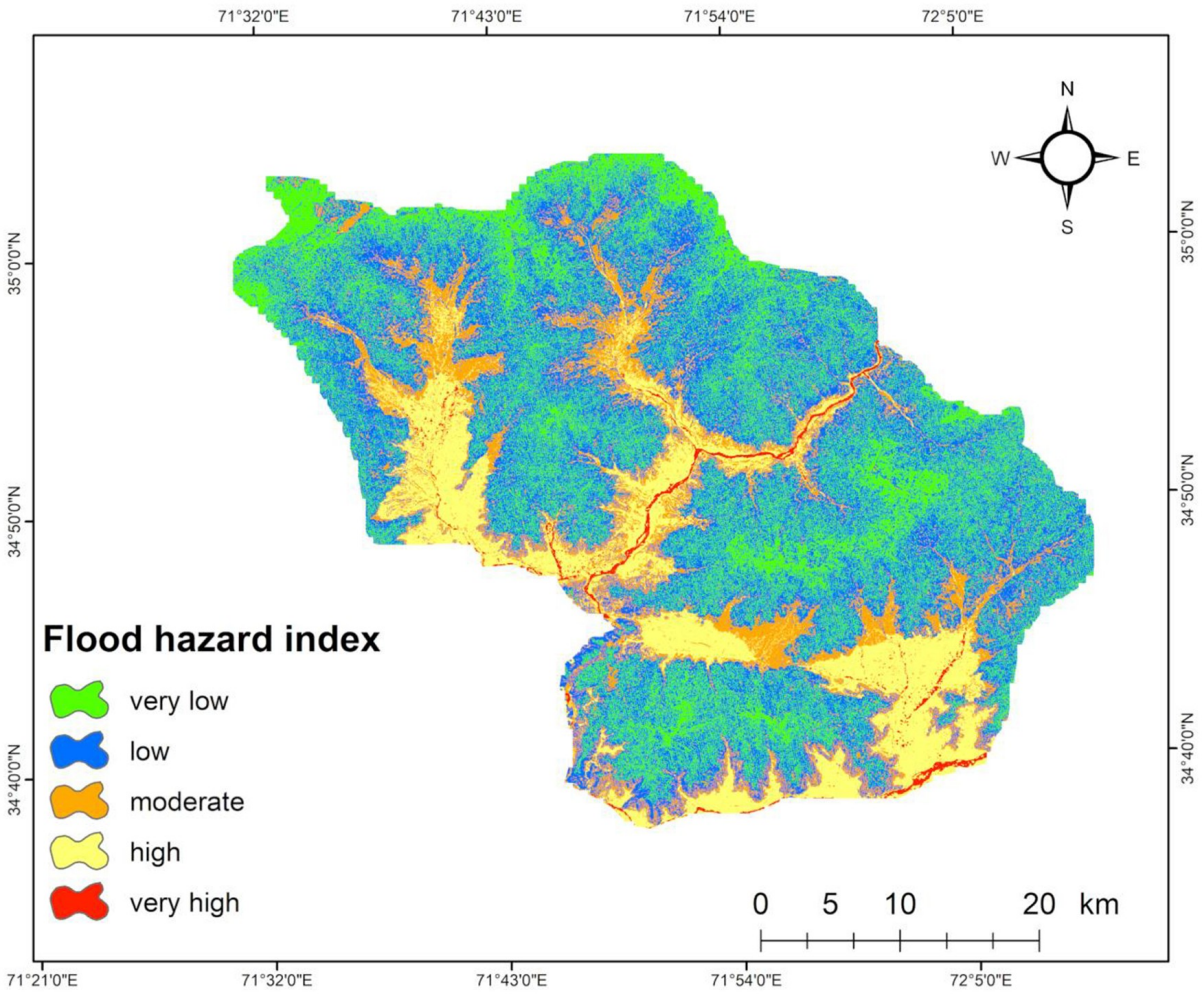
Flood hazard map of the study area.

In the study region, the slope has the maximum contribution to flooding with a PR value of 3.98 closely followed by LULC and elevation with PR values of 3.88, 3.41, respectively. The curvature, NDVI, and TWI have a medium influence on flood occurrence with PR values of 2.79, 1.92, and 1.81, respectively, while the drainage density and rainfall are the least important factors with PR values of 1.32 and 1.00, respectively, in determining flood susceptibility in the study region (Table 4). Fig 6 indicated that most of the very high and high risk areas are located near the banks of rivers Panjkora with low slope gradient, low elevation, flat curvature, higher TWI, and higher drainage density. From the final hazard map, it is clear that agriculture practices, commercial activities, or people living in high and very high flood susceptible zones are highly vulnerable to future flooding in the study region.

**Table 4:** Calculation results of weights for all conditioning factors.

Factor	Min RF	Max RF	(Max-Min)	Min total	PR(weight)
Elevation	0	0.56	0.56	0.19	3.41
Slope	0.02	0.68	0.66	0.19	3.98
Drainage density	0.02	0.24	0.22	0.19	1.32
Land use	0	0.64	0.64	0.19	3.88
Curvature	0.15	0.61	0.46	0.19	2.79
NDVI	0.11	0.43	0.32	0.19	1.92
TWI	0.08	0.38	0.30	0.19	1.81
Rainfall	0.11	0.28	0.17	0.19	1.00

### 3.1 Validation of flood hazard map

**Fig 7:**
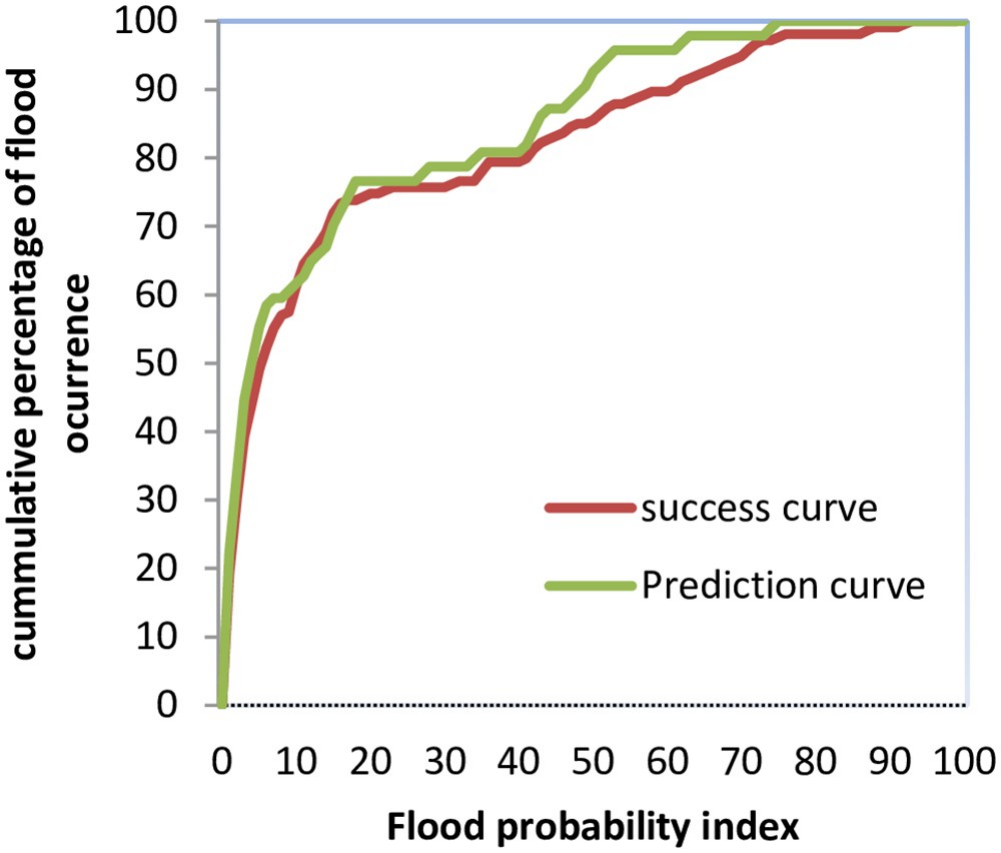
The ROC curve values of success rate and prediction rate.

The primary objective of hazard mapping is to demarcate the areas that are prone to flood hazards. There are many models used by researchers to analyze flood susceptibility, but it is essential to validate the results of the model used for flood hazard assessment [61,64]. The receiver operating characteristic (ROC) method is frequently used for the validation of prediction maps [9,53]. Moreover, the method is simple and produces clear and reliable results [25,65]. Many studies have used this method to validate results [1,26]. In this study, we used the ROC method to evaluate the success and prediction rate of the flood hazard map based on the previous flood incidents. To validate the model, we compared the existing flood data with the acquired flood probability map [64,66]. The results of the success rate were obtained using the training data set, and the prediction accuracy was calculated using the validation dataset that was not used in the training process [7,61,67]. The ROC curve for this study is shown in Fig 7, with AUC values of success and prediction accuracy of 82.04% and 84.74%, respectively.

## 4. Conclusion

Flood susceptibility mapping is an important step for future flood management. In hydrological and flood management studies, flood susceptibility maps are widely used to determine flood-prone zones. The present study aimed to assess flood hazards and map the flood-prone zones in the PRB, eastern Hindu Kush region. For this purpose, the RFR method was integrated with remote sensing and geospatial techniques to assess and map the flood hazard-prone areas. In this study, we used eight conditioning factors including slope, elevation, TWI, LULC, NDVI, drainage density, curvature, and rainfall to develop flood susceptibility maps. Overall, 154 flood-inundated locations were identified based on the damage and needs assessment report of the 2010 flood, field survey, interpretation of Landsat-7 and google earth images. The flood points were randomly divided into a training data set and testing data set. We used 70% (107 flood locations) of the points for building the model, and the remaining 30% (47 flood locations) points were employed in the validation of the probability model.

The flood hazard area was divided into five subclasses of hazard zones: very high, high, medium, low, and very low. The study found that approximately 15% of the total area is highly prone to flood hazard, 14% is moderately susceptible, 42% is low, and approximately 29% is very low. Furthermore, the study indicates that the high flood-prone areas are situated in the mid, southern, and western portions of the study area, as these areas are near the river with a low slope gradient, flat curvature, low elevation, high TWI value, and high drainage density. The ROC curve was used to measure the efficiency of the model and evaluate the results. The validation results showed good prediction efficiency with AUC values of success rate at 82.04% and of prediction rate at 84.74% of the flood susceptibility map. Therefore, the flood susceptibility map generated in this study can be considered an important tool to incorporate in flood risk management plans for disaster managers, decision-makers, and engineers. Based on the findings of this study, the concerned authorities can adopt appropriate mitigation and preparedness measures to minimize the impacts of prevailing and future floods.

## Supporting information

S1 Data (XLSX)

S2 Data (XLSX)

S3 Data (XLSX)

S1 File (RAR)
